# Osteopathic Manipulative Treatment in 564 Children with Congenital Heart Disease: A Project Report

**DOI:** 10.3390/children13020228

**Published:** 2026-02-05

**Authors:** Marco Petracca, Matteo Turinetto, Paola Sciomachen, Francesca Baroni, Christian Lunghi, Alessandro Accorsi, Mauro Longobardi, Ragini Pandey, Marco Pozzi

**Affiliations:** 1 Registry of Osteopaths of Italy, 20149 Milan, Italy; marcopetracca@me.com (M.P.); matteo.turinetto@isoi.it (M.T.); paola.sciomachen@gmail.com (P.S.); christian@bms-formation.com (C.L.); accorsiale@gmail.com (A.A.); mauro.longobardi@roi.it (M.L.); 2 Osteopathic Service, Osteobimbo Paediatric Clinic, 00188 Rome, Italy; 3 Neonatology and Pediatric Unit, San Pietro FBF Hospital, 00189 Roma, Italy; 4 Education Department of Osteopathy, Istituto Superiore di Osteopatia, 20126 Milan, Italy; 5 BMS Formation, 75116 Paris, France; 6 Department of Paediatric Cardiac Surgery, Sri Sathya Sai Sanjeevani Center for Child Heart Care, Atal Nagar (Naya Raipur) 492101, India; raginipandey07@gmail.com; 7 Department of Paediatric and Congenital Cardiac Surgery and Cardiology, Ospedali Riuniti, 60126 Ancona, Italy; mpozzi75@hotmail.com

**Keywords:** osteopathic medicine, osteopathic manipulation, osteopathic hospitals, pediatric intensive care units, congenital heart disease, length of stay, pain management, musculoskeletal pain, secondary prevention, tertiary prevention, quaternary prevention

## Abstract

**Highlights:**

**What are the main findings?**

- Osteopathic manipulative treatment can be safely integrated into multidisciplinary pediatric cardiac care.
- It may help improve the severity of somatic dysfunctions and reduce postoperative pain in children undergoing congenital heart disease surgery.

**What are the implications of the main findings?**

- Osteopathic care presents a promising adjunct to traditional pediatric cardiac treatments, particularly for improving musculoskeletal function and pain management.
- Further research is needed to assess the long-term outcomes and functional recovery of children undergoing congenital heart disease surgery with osteopathic care.

**Abstract:**

Background: Congenital heart diseases are the most common congenital malformations, affecting 4 to 9 per 1000 children, with increasing global prevalence. As surgical mortality rates decline, the focus has shifted toward improving the quality of life and perioperative outcomes for pediatric patients. Multidisciplinary rehabilitation, including osteopathic care, is increasingly incorporated into recovery programs. Osteopathic manipulative treatment combines manual techniques with lifestyle guidance to alleviate postoperative pain and promote recovery. This project report describes the impact of osteopathic manipulative treatment (OMT) on pain and somatic dysfunctions in hospitalized pediatric cardiac patients, using validated pain assessment tools. It presents a retrospective analysis of data collected as part of a humanitarian volunteer project. Methods: The project report follows a retrospective descriptive study design, using patient note forms from children aged 0–18 years undergoing cardiac surgery at the Sri Sathya Sai Sanjeevani Center in India between October 2023 and March 2024. A total of 29 experienced osteopaths recorded pain assessments at three time points—pre-surgery, post-surgery, and pre-discharge—using age-appropriate pain scales (FLACC, Wong-Baker Faces, and Numerical Rating Scale). Somatic dysfunctions were evaluated and classified using ICD-10 M99 codes. Data analysis involved descriptive statistics and pre-post comparisons using statistical software (Excel and OPENEPI). Results: The study included 564 children (60.5% male, mean age 5.8 ± 4.3 years). The most common congenital defects were ventricular septal defects (38.5%) and tetralogy of Fallot (21.6%). The average hospital stay was 15.9 ± 11.1 days. Significant reductions in pain scores were observed from the Intensive Care Unit to the postoperative ward (*p* < 0.001). Similarly, somatic dysfunction severity decreased significantly across hospitalization phases (*p* < 0.001). The thoracic region and rib cage were the most frequently affected areas. No adverse events related to osteopathic manipulative treatments were reported. Conclusions: This project report indicates that osteopathic manipulative treatment is safe and feasible for pediatric patients undergoing surgery for congenital heart disease. Pain scores and somatic dysfunction severity decreased during hospitalization. However, the lack of a control group, the heterogeneity of the patient population, and the short observation period limit the ability to draw causal conclusions. These findings provide a descriptive framework for integrating OMT into multidisciplinary pediatric cardiac care. Future studies should involve prospective, multicenter designs with control groups and longer follow-up periods to assess clinical, functional, developmental, and quality-of-life outcomes.

## 1. Introduction

Congenital heart diseases (CHDs) affect 4–9 per 1000 children at birth, representing the most common congenital malformations and a leading cause of infant morbidity and mortality worldwide [1]. Despite improvements in surgical outcomes and survival rates [2], children with CHD frequently experience significant perioperative morbidity, making quality of life and functional recovery increasingly important indicators of care quality [3,4]. Consequently, multidisciplinary rehabilitation programs have been advocated to address the complex medical, developmental, and psychosocial needs of this population [3,5]. Within this context, osteopathy (or osteopathic medicine) may represent a complementary therapeutic approach. Osteopathic care is defined as a patient-centered healthcare discipline that emphasizes the interrelationship between structure and function and supports the body’s innate self-regulatory and self-healing mechanisms, primarily through manual treatment [6]. Osteopathic manipulative treatment (OMT) addresses somatic dysfunctions (SD), understood as impaired or altered functions of the musculoskeletal, fascial, vascular, lymphatic, and neural systems [7]. Through the assessment and treatment of SD, OMT aims to influence regulatory processes and support overall physiological function [8,9]. In pediatric settings, OMT is often integrated with lifestyle and wellness guidance to promote adaptive capacity and development [10]. Preliminary evidence suggests that OMT may be beneficial as an adjunct to conventional care in postoperative contexts, potentially reducing pain and improving functional outcomes in both adult and pediatric populations [11,12,13]. This patient-centered approach aligns with osteopathic principles of supporting physical recovery while also addressing developmental, cognitive, and emotional needs [4,5,14]. In neonatal and preterm infants, OMT has been associated with reduced length of hospital stay and healthcare costs when included in multidisciplinary care programs [15,16,17]. Additionally, osteopathic interventions have been shown to reduce medication use and workplace absenteeism in individuals with musculoskeletal and functional disorders, suggesting potential broader social and economic benefits [18]. Moreover, when combined with other therapeutic modalities, osteopathic treatment proves beneficial not only for the individual but also for entire communities. A recent example of this is the contribution of osteopaths to improving health outcomes within underserved populations, as demonstrated in the case of the Haitian community [19]. However, despite growing interest in osteopathic care within pediatric management, evidence regarding its application in pediatric cardiac surgery—particularly in low-resource or humanitarian settings—remains limited and largely descriptive. To address this gap, from October 2023 to April 2024 the Registry of Osteopaths of Italy (ROI), in collaboration with the “Un Battito d’Ali” Association, implemented the humanitarian project “Mi Stanno a Cuore—They Are Close to My Heart” at the Sri Sathya Sai Sanjeevani Center for Child Heart Care in Chhattisgarh, India [20] (see Supplementary Material Video S1: Mi Stanno a Cuore: The Humanitarian Mission. Integrating Osteopathic Care into Sustainable Pediatric Cardiac Health Programs). This hospital provides free pediatric cardiac care, ensuring equitable access to diagnosis and surgical treatment regardless of socioeconomic status. Within this initiative, volunteer osteopaths supported the postoperative recovery of children undergoing cardiac surgery and provided interprofessional training to local healthcare staff. The present project report aims to provide a structured descriptive overview of this humanitarian experience and to explore the potential role of OMT in pediatric cardiac postoperative care. Specifically, we evaluated pain outcomes [21,22,23] and the presence of somatic dysfunctions [7], thereby contributing preliminary clinical data and practical insights into the integration of osteopathic care within sustainable pediatric cardiac health programs.

## 2. Materials and Methods

### 2.1. Design

This project report was designed as a retrospective descriptive study, incorporating elements of ecological study and clinical auditing [24]. Data were extracted from patient clinical records and subjected to descriptive analysis and pre-post treatment comparisons, following the methodological framework established by Aggarwal and Ranganathan [24] and previously validated study designs in pediatric research [25]. As this was a descriptive study based on the review of osteopathic note forms from a humanitarian voluntary project, ethical approval was not required. The study adhered to the ethical principles outlined in the Declaration of Helsinki and complied with the National Ethical Guidelines for Biomedical and Health Research Involving Human Participants issued by the Indian Council of Medical Research (2017), which qualifies the research for an exemption from formal ethics committee review [26]. This exemption was granted due to the retrospective nature of the analysis, using de-identified archival and secondary data originally collected for routine clinical care. No new interventions or patient interactions were introduced for the research, and all data were anonymized before analysis to ensure participant confidentiality. Administrative authorization for the retrieval of anonymized clinical data was obtained from the Institutional Board of the Registry of Osteopaths of Italy (ROI), the organizing entity for the humanitarian project. These records were securely stored in a password-protected electronic repository to ensure maximum data protection. Informed consent for hospital procedures was provided by the parents or caregivers of all participants.

### 2.2. Sample

Patient note forms were retrospectively reviewed to extract data on children aged 0–18 years admitted for cardiac surgery at the Sri Sathya Sai Sanjeevani Center for Child Heart Care in Atal Nagar (Naya Raipur), Chhattisgarh, India. These patients were enrolled in the humanitarian project, involving volunteer osteopaths, “Mi stanno a Cuore—They are close to my heart”, between October 2023 and March 2024 [20]. Patients with severe postoperative complications, serious comorbidities, or without explicit consent to the procedures were excluded.

### 2.3. Practitioners

29 Italian volunteer experienced osteopaths [27] were selected by the ROI for this humanitarian project to perform data collection, pain assessments, and osteopathic evaluations and treatments in collaboration with the hospital team. The participating osteopaths underwent a curriculum-based training program aligned with the Benchmark for Training in Osteopathy issued by the World Health Organization [28]. All volunteers participated in a 16 h training session in Milan, Italy, where a professional consensus was established to ensure a unified approach consistent with the core competencies in osteopathy as outlined by ROI [29], thereby strengthening consistency across assessments and treatments.

### 2.4. Outcome Measures

To provide a descriptive framework of this project, volunteer osteopaths collected data on children’s hospital course and characteristics, including: Number of treated patients; Gender; Age; Type of delivery; Gestational age; Weight at birth; Parity; Diagnosis; Type of surgery; LOS (Date of admission—Days in intensive care unit (ICU)—Days in step down—Days in post op). Three algometric scales were also used by hospital nurses to assess pain progression, based on their suitability, feasibility, and applicability for measuring pain in children aged 0 to 18 years. The FLACC scale was implemented for children under 3 years of age, or for children who, due to motor or cognitive deficits, cannot provide a subjective assessment of pain [21,30]. The scale with Wong-Baker Faces was administered to children aged > 3 years [31], while the Numerical rating scale was used for children aged ≥ 8 years [24].

While these tools utilize different psychometric approaches (behavioral observation vs. self-report), their integration into a unified analysis is justified by their standardized 0–10 scoring metric, which ensures functional comparability in a real-world clinical context. This multi-tool approach is crucial in heterogeneous pediatric cohorts to guarantee that pain is measured with the most developmentally appropriate and validated instrument for each age group. To ensure clarity and consistency, evaluations were conducted at three key time points: pre-operative, post-operative, and pre-discharge. At each of these phases, osteopaths recorded both SD and pain scores of the age-appropriate scales. This approach allows a comprehensive understanding of SD patterns and pain trajectories throughout hospitalization. In particular, the volunteer osteopaths recorded the SD detected during the first pre-operative evaluation, in the first post-operative evaluation, and in the last treatment in the post op/pre-discharge ward. SD were coded using the severity score classification reported in the Outpatient Osteopathic SOAP Note Form [32] and ICD-10 M99 codes [33], which allow a standardized description of osteopathic findings across 11 anatomical regions. This system has been previously implemented in pediatric and neonatal OMT research [15,34]. Every event or adverse reaction during OMT was recorded.

### 2.5. Procedures

During the study period, data collection, evaluations, and OMT were started in the pre-surgical phase, when the child was admitted to the hospital, after the opening of the medical record. The pain scale and assessments/treatments were administered 3 times: pre-surgery, post-surgery, and before discharge. The volunteer osteopaths carried out all the osteopathic assessments/treatments and, with the help of the hospital healthcare nurses who administered the pain scales, transcribed the data on the Excel sheet of the computer. Each OMT session involved an osteopathic examination and personalized osteopathic approaches used in pediatric settings [35,36]. The osteopathic evaluation was usually performed with the child lying down in their bed. Diagnostic criteria for SD were based on the parameters previously described in the osteopathic glossary, focusing on the TART components: Tissue texture abnormalities, Asymmetry of structure, Restriction of motion, and Tenderness [7]. The volunteer osteopaths recorded the regions and the intensity of the detected SD on the Excel sheet. According to the ICD-10 codes [33], the Regions associated with SD are: (1) Head, (2) Cervical, (3) Thoracic, (4) Lumbar, (5) Sacrum, (6) Pelvis, (7) Lower Extremities, (8) Upper Extremities, (9) Rib Cage, (10) Abdomen, (11) Other (Systemic SD or General SD, or involving multiple regions). The evaluation brings for each of these 11 regions a severity score ranging from 0 to 3 and allows to obtain a minimum score of 0 and a maximum score of 33, as described in the Outpatient Osteopathic SOAP Note Form [32], and implemented in the OSCAR score used by Hery et al. [11]: 0: no SD; 1: mild or questionable SD; 2: present and moderate SD; 3: severe SD; N/A: not assessable. The OMT procedures employed a dual strategy that integrated personalized interventions with standardized frameworks, facilitated by the implementation of a decision-making framework designed to guide clinical reasoning in complex scenarios [37,38]. The first phase adhered to the standardized clinical protocol outlined by Roland et al. [39], which was adapted to the specific operational requirements of each hospital ward (Table 1) and tailored to the age-specific needs of the pediatric population. These standardized techniques were performed exclusively based on the patient’s clinical tolerance, ensuring safety in high-acuity settings.

Building upon this baseline, the second phase focused on interventions tailored to the specific SD identified during assessment, aiming to enhance pain management, range of motion, and functional outcomes such as respiration, digestion, and mobility [35,36]. This stage involved a personalized treatment plan developed according to the Five Osteopathic Models (i.e., biomechanical, neurological, respiratory-circulatory, metabolic, and behavioral) (Table 2) [38]. Both maximalist and minimalist approaches were employed—selecting from established pediatric osteopathic methodologies [6,7,28,36,39]—and further refined based on each patient’s clinical status, functional profile, and tolerance [10,37,38]. For example, within the respiratory-circulatory model, techniques and SD-associated areas were selected to evoke improvements in respiratory patterns, oxygen saturation, and heart rate variability. Each session lasted between 15 and 45 min, with the duration adjusted according to the patient’s functional profile. For example, consistent with the behavioral model, specific techniques and target areas were chosen to mitigate stress behaviors and improve autonomic regulation. This dual-layer methodology ensures methodological reproducibility through a shared procedural foundation while maintaining the diagnostic flexibility essential for managing complex pediatric cardiac cases.

### 2.6. Statistical Analysis

For all collected data, a quantitative analysis was performed using means, standard deviations, and frequencies (ratios and proportions) with the Excel sheet. Statistical programs such as Excel and OPENEPI version 3.01 were used for pre-post treatment comparisons and analyses. The original data supporting the findings of this retrospective descriptive study are available upon request from the corresponding author, subject to approval by the Registry of Osteopaths of Italy.

### 2.7. Data Integrity and Bias Mitigation

To ensure the objectivity of the findings and mitigate potential institutional bias, several safeguards were implemented. First, all clinical data collected during the humanitarian mission were anonymized and de-identified at the source before being transferred for analysis. Second, the statistical processing and interpretation of the results were performed by researchers using blinded datasets, ensuring that the analysis remained independent of the funding body’s organizational roles. Furthermore, the study followed a pre-defined clinical audit protocol based on the methodological framework for descriptive research [24], which prioritizes standardized reporting over subjective interpretation.

## 3. Results

### 3.1. Sample Characteristics

A total of 564 children (60.5% male, 39.5% female) aged between 1 month and 18 years (mean age 5.8 ± 4.3 years) were included in the study. The age distribution showed that 7.2% of patients were under 1 year, 35.4% were between 1 and 3 years, 22.6% between 4 and 6 years, 26% between 7 and 12 years, and 9% were older than 13 years (Table 3).

### 3.2. Diagnosis and Length of Stay

The most frequent CHD were ventricular septal defects (38.5%), followed by tetralogy of Fallot (21.6%) and atrial septal defects (19.2%). Less common diagnoses included right ventricular outflow tract obstruction (5.5%), patent ductus arteriosus (5%), double-chambered right ventricle (3.7%), partial anomalous pulmonary venous connection (2.3%), coarctation of the aorta (2.1%), total anomalous pulmonary venous connection (1.4%), and atrioventricular canal defect (0.7%). The overall LOS ranged from 6 to 76 days, with a mean of 15.9 ± 11.1 days. Patients spent an average of 3.3 ± 2.5 days in the ICU, 1.6 ± 1.8 days in the step-down unit, and 2.3 ± 2 days in the postoperative ward (Table 4).

### 3.3. Pain Assessment

Pain assessment using FLACC, Wong-Baker Faces, and Numerical Rating Scales demonstrated that mean pain scores were 0.9 ± 1.5 preoperatively, increased to 3.3 ± 2.7 during ICU stay, decreased to 2.4 ± 2.4 in the step-down unit, and further decreased to 1.4 ± 1.9 in the postoperative ward. The reduction in pain scores from the ICU to the postoperative ward was statistically significant (*p* < 0.001) (Table 5).

A total of 1655 osteopathic assessments and treatments were delivered by 29 volunteer osteopaths over 188 days. Severity scores, reflecting the overall SD burden, were 5.8 ± 6.3 preoperatively, 10.1 ± 7.7 during ICU stay, 7.1 ± 6.5 in the step-down unit, and 5.5 ± 5.8 in the postoperative ward. The decreases observed from ICU to step-down and postoperative phases were statistically significant (*p* < 0.001) (Table 6). The regions of the body framework frequently associated with SD were the thoracic and the rib cage, in all of the hospitalization phases.

However, during LOS, SD was widespread in the whole body, affecting multiple regions. No adverse events or reactions related to OMT were reported during the treatment itself.

## 4. Discussion

This project report represents the first large-scale descriptive analysis evaluating the application of OMT in children undergoing surgical correction for CHD. A total of 564 pediatric patients were enrolled, and 29 volunteer osteopaths administered 1655 osteopathic sessions as part of a six-month humanitarian project. The data, retrospectively collected from patient note forms, offer a comprehensive descriptive overview of SD patterns across different phases of hospitalization, pain intensity, and the feasibility of integrating OMT within a multidisciplinary pediatric cardiac care program. The distribution and intensity of SD were recorded across different hospitalization phases through the severity score. Comprehensively, osteopathic evaluations indicated that SD were most frequently observed in the thoracic region and rib cage. These observations highlight patterns of SD presentation in children undergoing cardiac surgery [11,13]. The statistically significant finding is that, overall, SD improved from ICU to step down/post-op LOS, with a reduction in both the severity score and the number of SD. Pain assessments measured using the FLACC, Wong-Baker Faces, and Numerical Rating Scales showed higher scores during the ICU stay compared to pre-operative evaluations, with subsequent decreases in the step-down unit and postoperative ward. These data describe the temporal trajectory of pain experienced by pediatric patients during hospitalization, providing a descriptive overview of pain variations across perioperative phases without implying causal relationships with osteopathic care [40,41,42]. Non-pharmacological interventions such as therapeutic touch, massage therapy, or music-assisted hand massage have also been associated with reductions in pain and anxiety in pediatric hospital settings [43,44]. In this context, OMT may represent an additional therapeutic approach within multimodal pediatric care programs [45]. The data also provide descriptive insights into hospital LOS, intensive care unit duration, step-down unit duration, and postoperative recovery timelines, which together contribute to a broader understanding of the clinical course in children with CHD.

The LOS observed in our cohort (mean 15.9 ± 11.1 days) is consistent with international reports on pediatric cardiac surgery [46,47]. However, socioeconomic factors and hospital-specific determinants are known to influence patient discharge, highlighting the need for controlled, multicenter studies to disentangle the role of osteopathic care within broader determinants of recovery [48,49]. Future controlled studies could explore whether the integration of OMT, as part of multidisciplinary care, may contribute to influencing hospital recovery trajectories, including LOS [50]. The most common diagnoses were ventricular septal defects, tetralogy of Fallot, and atrial septal defects, reflecting the typical prevalence distribution observed in this population [1,2]. No adverse events or reactions related to OMT were reported, suggesting that the interventions were well-tolerated in this patient population. Our findings on feasibility and safety align with previous evidence in neonatal and pediatric populations, where OMT was shown to reduce LOS and costs without adverse events. In particular, Lanaro et al. [15] and Cicchitti et al. [51] observed that OMT can be integrated in intensive care settings with positive outcomes on hospitalization parameters. More recently, Vismara et al. [34] correlated SD severity with vagal cardiac tone in preterm infants, supporting the physiological plausibility of OMT in vulnerable populations. From an observational standpoint, this finding contributes to the descriptive safety profile of osteopathic interventions in children undergoing cardiac surgery. However, no standardized method for assessing adverse events or reactions was applied, which represents a limitation to this statement. The OMT administered to newborns by the volunteer osteopaths involved in the project is consistent with the approaches described by Wernick and Berkowitz (2019) [52], and the routine reported by Roland et al. [39], which is implemented in the Neonatal Intensive Care Unit (NICU) to reduce hospital stays and healthcare costs. Furthermore, the osteopathic approaches employed by the volunteer osteopaths in this hospital setting with pediatric patients align with those reported by a panel of Italian osteopathic practitioners [53]. These approaches emphasize an interprofessional attitude, person-centered osteopathic practice, evidence-informed care, and methods grounded in osteopathic principles and models, all of which guide treatment toward supporting the functional profile of the pediatric patient [53]. In the context of multidisciplinary pediatric cardiac care, the present study offers a descriptive framework for documenting osteopathic involvement. Patterns of SD, pain trajectories, and treatment frequency provide valuable descriptive data for understanding the integration of osteopathic care within a hospital setting. While causal relationships cannot be inferred, these descriptive findings may inform the design of future hypothesis-driven studies, including prospective observational studies or controlled trials, aimed at exploring potential associations between osteopathic care and clinical or functional outcomes. Finally, this study illustrates a possible approach for collecting detailed observational data in pediatric populations with complex clinical conditions. A growing trend in surgical care is the investigation and incorporation of multimodal interventions into standardized programs; in this context, manual therapies such as OMT are increasingly being used in the management of patients requiring surgical procedures [13,54]. The descriptive dataset presented herein can support the development of evidence-informed approaches for documentation and assessment of osteopathic care within multidisciplinary care pathways.

### 4.1. Limitations

Despite the positive trends observed in our cohort, the findings must be interpreted within the broader context of the current pediatric manual medicine literature, which remains a subject of academic debate. While our data align with several clinical observations, high-impact systematic reviews—most notably by Posadzki et al. in the journal Pediatrics [55]—have critically pointed out that much of the evidence supporting OMT in children is limited by methodological flaws and a high risk of bias.

Recent syntheses of manual therapy, including the work of De Marsh et al. (2021) [56] and Tedeschi and Giorgi (2025) [57], further emphasize that while OMT appears safe and potentially beneficial for functional disorders and pain modulation, the lack of rigorous, large-scale randomized controlled trials prevents definitive conclusions regarding its specific efficacy. These authors highlight a predominance of small-sample studies and the potential for publication bias, where neutral or negative results may remain underreported. By acknowledging these constraints, this study does not aim to provide conclusive proof of efficacy; rather, it seeks to address the gap identified by the wider medical community for transparent, large-scale reporting. This clinical audit serves as a contribution to ‘real-world’ evidence, providing a foundational safety and feasibility profile that is often missing from the highly controlled but small-sample literature typically found in major pediatric or cardiology journals. This study presents several limitations that should be acknowledged. First, its descriptive design, without the inclusion of a control group, does not allow any causal inference regarding the relationship between OMT and changes in pain or reduction in LOS. In addition, the single-center setting limits the generalizability of the findings. Second, pain assessment scores, although conducted using validated scales such as the FLACC, Wong-Baker Faces, and numerical rating scale, were analyzed collectively, which may have influenced the overall interpretation. The procedures implemented in this study involve the subjectivity in the scoring of SD, as assessments were carried out by a group of 29 osteopaths. Despite shared training and the establishment of a professional consensus, some variability in the interpretation and scoring of SD may have arisen across practitioners, which we acknowledge as an inherent limitation of the study. An important limitation of this study is the lack of a standardized method for assessing and recording adverse events or reactions during OMT. While no adverse events were reported, the absence of a formalized assessment system limits the ability to fully evaluate the safety of OMT in this pediatric population. Another important limitation relates to the heterogeneity of the study population: pediatric patients with CHD undergo different surgical procedures and present diverse comorbidities and postoperative trajectories, making stratification and comparison across subgroups challenging. Furthermore, the observation period was restricted to hospitalization, without extending follow-up to medium- or long-term outcomes, such as recovery trajectories, quality of life, or neurodevelopmental progress. Finally, it should be considered that the patients were managed within a multidisciplinary care pathway, where multiple interventions were provided simultaneously. This multimodal context prevents disentangling the specific contribution of osteopathic care from the effects of other therapeutic components. Similar challenges are reported in other pediatric OMT studies, as highlighted in a recently published scoping review [56]. These limitations underscore the need for well-designed, multicenter studies with long-term follow-up that integrate clinical outcomes, neurodevelopmental measures, and quality-of-life assessments. In conclusion, while we acknowledge the risk of ecological fallacy inherent in descriptive designs [24], the consistency of data across a large cohort of 564 individuals minimizes the likelihood of chance observations.

### 4.2. Future Directions

Future research should incorporate both qualitative and quantitative approaches to address the limitations of this study and to further advance the understanding of osteopathic care in pediatric cardiac units/settings (Table 7). Based on the descriptive findings of the present study, future controlled research should explicitly test the hypothesis that osteopathic care contributes to improvements in functional recovery, developmental trajectories, and quality-of-life outcomes in pediatric cardiac patients. Qualitative research will be crucial to explore the attitudes, preferences, and experiences of osteopaths involved in the study, particularly regarding osteopathic assessment and treatment.

These findings will help clarify how clinical reasoning and hands-on approaches are currently applied in this setting and will inform the development of a future osteopathic intervention protocol grounded in practitioners’ direct experience. In parallel, qualitative data collected from patients and caregivers will provide essential insights into their perceptions, expectations, and lived experiences of osteopathic care, supporting the development of patient-centered interventions aligned with their needs. Building on these qualitative insights, future quantitative studies should be designed to test specific, a priori hypotheses, including whether osteopathic care is associated with measurable improvements in functional outcomes, developmental parameters, and health-related quality of life. Longer-term follow-up studies will be particularly important to evaluate the sustainability of these potential benefits over time. To enhance methodological rigor, standardized training and calibration of osteopaths performing assessments should be implemented to reduce subjectivity and improve inter-rater reliability. Furthermore, stratification of patients according to surgical procedures and clinical characteristics will allow for hypothesis-driven analyses of differential effects across subgroups, thereby identifying priority clinical and functional outcomes for targeted interventions. Finally, prospective, multicenter studies with appropriate sample sizes and control or comparator groups should be conducted to rigorously evaluate both the efficacy and effectiveness of osteopathic care. Inclusion of multiple regional hospitals where integrative osteopathic care is practiced alongside standard treatments will strengthen external validity and support the development of a standardized osteopathic care protocol applicable across diverse clinical settings.

## 5. Conclusions

This project report describes a study that shows the feasibility and safety of OMT in children undergoing surgical correction for congenital heart disease (CHD). A total of 564 pediatric patients received 1655 OMT sessions delivered by 29 volunteer osteopaths over 188 days. Postoperative pain scores decreased from the ICU to the postoperative ward (*p* < 0.001), and severity scores, reflecting the burden of somatic dysfunction (SD), also showed a significant reduction across hospitalization phases (*p* < 0.001). No adverse events or reactions were reported during the OMT sessions. These findings provide a descriptive overview of the integration of OMT into multidisciplinary pediatric cardiac care. They offer preliminary descriptive data that can inform future prospective studies aimed at evaluating functional and clinical outcomes, recovery trajectories, and quality of life in this patient population.

## Figures and Tables

**Table 1:** Phase 1: Standardized clinical protocol.

OMT (Method)	Description	OMT (Technique)
Lymphatic Pump Technique (LPT)	Uses a sequential pneumatic compressive pumping action to reduce swelling and enhance immune function by mimicking the lymphatic system.	Thoracic pump technique
Diaphragm Treatment (DPT)	Aims to improve a dysfunctional thoracic diaphragm and optimize its function, affecting the thoracic and lumbar spine, ribs, and sternum.	Functional method
Balanced Ligamentous Tension (BLT)	Manipulates joint ligaments to a precise balance point, equalizing stress in all directions, minimizing tension.	Rib raising technique (pressure applied on rib angles)
Myofascial Release (MFR)	A category of techniques aimed at releasing muscle and fascia restrictions. Targets flexion, extension, and side bending limitations, improving the restrictive barrier.	Thoracic inlet technique
Osteopathic Cranial Manipulation (OCM)	Primarily uses respiratory mechanisms and balanced membranous tension to treat cranial-related somatic dysfunctions.	Occipital release technique

**Table 2:** Phase 2. Personalized Treatment Plan Based on the Five Osteopathic Models.

Osteopathic Model	Functional Profile Pattern *
Biomechanical	Improve mobility, range of motion, and alignment
Neurological	Enhance neurological function, reduce tension and improve sensory processing
Respiratory-Circulatory	Improve respiratory patterns, oxygen saturation, and heart rate variability
Metabolic	Diminished tension and abdominal allodynia
Behavioral	Mitigate stress behaviors, improve autonomic regulation, and emotional balance

* Session duration and type of technique are selected based on variations in the functional profile pattern, ensuring individualized treatment tailored to the patient’s clinical condition.

**Table 3:** Sample Characteristics.

Characteristics	Value
Sample (*n*)	564
Male/Female (%)	60.5/39.5
Age (Y; M ± SD)	5.8 ± 4.3 (Range: 1 m–18 y)
**Age Distribution**	**%**
0–11 months	7.2
1–3 years	35.4
4–6 years	22.6
7–12 years	26
>13 years	9

**Table 4:** Diagnosis and length of stay.

Diagnosis	%
Ventricular Septal Defect	38.5
Tetralogy of Fallot	21.6
Atrial Septal Defect	19.2
Right ventricular outflow tract	5.5
Patent Ductus Arteriosus	5
Double-chambered right ventricle	3.7
Partial Anomalous Pulmonary Venous Connection	2.3
Coarctation of the Aorta	2.1
Total Anomalous Pulmonary Venous Connection	1.4
Atrial Ventricular Canal Defect	0.7
**Length Of Stay (LOS)**	**DAYS**
Range	6–76
Total (M ± SD)	15.9 ± 11.1
ICU	3.3 ± 2.5
Step down	1.6 ± 1.8
Post-op	2.3 ± 2

**Table 5:** Pain assessment.

Hospitalization Phase	Pain Scale	M ± SD	Range
Pre op	FLACC-FACES-NRS	0.9 ± 1.5	Range 0–8
ICU	FLACC-FACES-NRS	3.3 ± 2.7	Range 0–10
Step down	FLACC-FACES-NRS	2.4 ± 2.4	-
Post-op	FLACC-FACES-NRS	1.4 ± 1.9 *	-

* Pain in Post-op LOS significantly decreases from ICU LOS (*p* < 0.001).

**Table 6:** Osteopathic Assessments and Treatment and Distribution of Somatic Dysfunctions.

Description	Value	
Assessments/treatments (*n*)	1655	
Volunteer osteopaths (*n*)	29	
Period (9 October 2023–14 April 2024)	188 days	
**Severity Score (mean ± SD, range)**		
Hospitalization Phase	Severity Score	Range
Severity score Pre op	5.8 ± 6.3	(0–26)
Severity score ICU	10.1 ± 7.7	(0–33)
Severity score Step down	7.1 ± 6.5	(0–25) *
Severity score Post op	5.5 ± 5.8	(0–29) *

* The mean severity score and the length of stay in the Step-down and Post op were significantly lower than those in the ICU (*p* < 0.001).

**Table 7:** Research road map.

Research Objectives	Study Type
Evaluate the attitudes and preferences of osteopaths involved in the study regarding osteopathic assessment and treatment. Inform the development of a future osteopathic intervention protocol based on the direct experience of practitioners in this specific setting	Qualitative Study (Focus Group)
Collect data on the experiences of patients and caregivers regarding osteopathic care in pediatric cardiac rehabilitation	Qualitative Study (Interviews/Patient Surveys/Descriptive Phenomenological Study)
Incorporate control groups or comparator interventions to evaluate the specific effects of osteopathic care	Randomized Controlled Trial (RCT)
Conduct longer-term follow-up to assess functional, developmental, and quality-of-life outcomes	Longitudinal Cohort Study or Follow-up Study
Standardize training and calibration of osteopaths performing assessments to reduce subjectivity	Educational Intervention Study + Inter-rater Reliability Testing
Stratify patients by surgical procedure and characteristics for more nuanced insights	Stratified Analysis within a Cohort Study
Design prospective, multicenter studies with appropriate sample sizes	Multicenter, Prospective Cohort Study

## Data Availability

The original data supporting the findings of this retrospective descriptive study are available upon request from the corresponding author, subject to approval by the Registry of Osteopaths of Italy.

## Supplementary Materials

The following supporting information can be downloaded at: https://www.mdpi.com/article/10.3390/children13020228/s1, Video S1: Mi Stanno a Cuore: The Humanitarian Mission. Integrating Osteopathic Care into Sustainable Pediatric Cardiac Health Programs.

## Abbreviations

The following abbreviations are used in this manuscript:

CHDs	Congenital Heart Diseases
OMT	Osteopathic Manipulative Treatment
SD	Somatic Dysfunction
LOS	Length Of Stay
ICU	Intensive Care Unit
ROI	Registry of Osteopaths of Italy

## References

[1] van der Linde D., Konings E.E., Slager M.A., Witsenburg M., Helbing W.A., Takkenberg J.J., Roos-Hesselink J.W. (2011). Birth prevalence of congenital heart disease worldwide: A systematic review and meta-analysis. J. Am. Coll. Cardiol..

[2] Liu Y., Chen S., Zühlke L., Black G.C., Choy M.K., Li N., Keavney B.D. (2019). Global birth prevalence of congenital heart defects 1970–2017: Updated systematic review and meta-analysis of 260 studies. Int. J. Epidemiol..

[3] Ubeda Tikkanen A., Vova J., Holman L., Chrisman M., Clarkson K., Santiago R., Schonberger L., White K., Badaly D., Gauthier N. (2023). Core components of a rehabilitation program in pediatric cardiac disease. Front. Pediatr..

[4] Ubeda Tikkanen A., Oyaga A.R., Riaño O.A., Álvaro E.M., Rhodes J. (2012). Paediatric cardiac rehabilitation in congenital heart disease: A systematic review. Cardiol. Young.

[5] Ubeda Tikkanen A., Berry E., LeCount E., Engstler K., Sager M., Esteso P. (2021). Rehabilitation in pediatric heart failure and heart transplant. Front. Pediatr..

[6] European Committee for Standardisation (CEN) (2015). European Standard on Osteopathic Healthcare Provision.

[7] American Association of Colleges of Osteopathic Medicine (2017). Glossary of Osteopathic Terminology.

[8] Consorti G., Castagna C., Tramontano M., Longobardi M., Castagna P., Di Lernia D., Lunghi C. (2023). Reconceptualizing somatic dysfunction in the light of a neuroaesthetic enactive paradigm. Healthcare.

[9] Lunghi C., Baroni F., D’Alessandro G., Consorti G., Tramontano M., Stubbe L., Conte J., Liem T., Zegarra-Parodi R. (2025). Patient–practitioner–environment synchronization: Four-step process for integrating interprofessional and distinctive competencies in osteopathic practice—A scoping review with integrative hypothesis. Healthcare.

[10] Lunghi C., Baroni F., Amodio A., Consorti G., Tramontano M., Liem T. (2022). Patient active approaches in osteopathic practice: A scoping review. Healthcare.

[11] Hery M., Manscourt C., Bajolle F., Renaudo S., Soudain-Pineau M., Bonnet D., Stubbe L. (2020). Effets d’une prise en charge ostéopathique sur les douleurs postopératoires de nouveau-nés et nourrissons opérés d’une cardiopathie congénitale: Essai clinique randomisé en simple aveugle. Rev. Osteopathie.

[12] Piccolo R.L., Cantagalli M.M., Ferroni T., Fracchiolla F., Morabito A. (2025). The Effect of Osteopathic Manipulative Treatment on Length of Stay and Pain Relief in Pediatric Appendectomy: A Pilot Non-Randomized Time-Controlled Clinical Trial. Front. Pediatr..

[13] Rorris F.P., Skouteli E.A.T., Papakonstantinou K., Kokotsaki L., Skotiniotis E., Kokotsakis J. (2022). Osteopathic manipulative treatment in cardiac surgery patients: A systematic review. Int. J. Osteopath. Med..

[14] Cassidy A.R., Butler S.C., Briend J., Calderon J., Casey F., Crosby L.E., Fogel J., Gauthier N., Raimondi C., Marino B.S. (2021). Neurodevelopmental and psychosocial interventions for individuals with CHD. Cardiol. Young.

[15] Lanaro D., Ruffini N., Manzotti A., Lista G. (2017). Osteopathic manipulative treatment showed reduction of length of stay and costs in preterm infants: A systematic review and meta-analysis. Medicine.

[16] Cerritelli F., Pizzolorusso G., Ciardelli F., La Mola E., Cozzolino V., Renzetti C., D’iNcecco C., Fusilli P., Sabatino G., Barlafante G. (2013). Effect of osteopathic manipulative treatment on length of stay in a population of preterm infants: A randomized controlled trial. BMC Pediatr..

[17] Cerritelli F., Pizzolorusso G., Renzetti C., Cozzolino V., D’Orazio M., Lupacchini M., Marinelli B., Accorsi A., Lucci C., Lancellotti J. (2015). A multicenter randomized controlled trial of osteopathic manipulative treatment on preterms. PLoS ONE.

[18] Price J.W. (2021). Osteopathic model of the development and prevention of occupational musculoskeletal disorders. J. Osteopath. Med..

[19] Coupet S., Howell J.D., Ross-Lee B. (2013). An international health elective in Haiti: A case for osteopathic medicine. J. Am. Osteopath. Assoc..

[20] Registry of Osteopaths of Italy. Mi Stanno a Cuore. https://www.registro-osteopati-italia.com/mi-stanno-a-cuore/.

[21] Merkel S.I., Voepel-Lewis T., Shayevitz J.R., Malviya S. (1997). The FLACC: A behavioral scale for scoring postoperative pain in young children. Pediatr. Nurs..

[22] Tsze D.S., von Baeyer C.L., Bulloch B., Dayan P.S. (2013). Validation of self-report pain scales in children. Pediatrics.

[23] Miró J., Castarlenas E., Huguet A. (2009). Evidence for the use of a numerical rating scale to assess intensity of pediatric pain. Eur. J. Pain.

[24] Aggarwal R., Ranganathan P. (2019). Study designs: Part 2–descriptive studies. Perspect. Clin. Res..

[25] Panza R., Piarulli F., Rizzo V., Schettini F., Baldassarre M.E., Di Lorenzo A., Tafuri S., Laforgia N. (2024). Positional plagiocephaly: Results of the osteopathic treatment of 424 infants. Ital. J. Pediatr..

[26] Indian Council of Medical Research National Ethical Guidelines for Biomedical and Health Research Involving Human Participants. https://www.indiascienceandtechnology.gov.in/sites/default/files/file-uploads/guidelineregulations/1527507675_ICMR_Ethical_Guidelines_2017.pdf.

[27] Ericsson K.A., Prietula M.J., Cokely E.T. (2007). The making of an expert. Harv. Bus. Rev..

[28] World Health Organization (2010). Benchmarks for Training in Osteopathy. https://www.who.int/publications/i/item/9789241599665.

[29] Sciomachen P., Arienti C., Bergna A., Castagna C., Consorti G., Lotti A., Lunghi C., Tramontano M., Longobardi M. (2018). Core competencies in osteopathy: Italian register of osteopaths proposal. Int. J. Osteopath. Med..

[30] Peng T., Qu S., Du Z., Chen Z., Xiao T., Chen R. (2023). A systematic review of the measurement properties of Face, Legs, Activity, Cry, and Consolability scale for pediatric pain assessment. J. Pain Res..

[31] Wong-Baker Faces Foundation Wong-Baker FACES^®^ Pain Rating Scale. https://wongbakerfaces.org/.

[32] Sleszynski S.L., Glonek T., Kuchera W.A. (1999). Standardized medical record: Validation of a standardized osteopathic SOAP note. J. Am. Osteopath. Assoc..

[33] World Health Organization ICD-10 Version 2019: Somatic Dysfunction M99.0. https://icd.who.int/browse10/2019/en#/M99.0.

[34] Vismara L., Gianmaria Tarantino A., Bergna A., Bianchi G., Bragalini C., Billò E.D., Farra F.D.D., Buffone F.D., Agosti M. (2022). Correlation between diminished vagal tone and somatic dysfunction severity in very and extremely low birth weight preterm infants assessed with frequency spectrum heart rate variability and salivary cortisol. Medicine.

[35] Parnell Prevost C., Gleberzon B., Carleo B., Anderson K., Cark M., Pohlman K.A. (2019). Manual therapy for the pediatric population: A systematic review. BMC Complement. Altern. Med..

[36] Bagagiolo D., Didio A., Sbarbaro M., Priolo C.G., Borro T., Farina D. (2016). Osteopathic manipulative treatment in pediatric and neonatal patients and disorders: Clinical considerations and updated review of the existing literature. Am. J. Perinatol..

[37] Lunghi C., Baroni F. (2019). Cynefin framework for evidence-informed clinical reasoning and decision-making. J. Am. Osteopath. Assoc..

[38] Castagna C., Consorti G., Turinetto M., Lunghi C. (2021). Osteopathic models integration radar plot: A proposed framework for osteopathic diagnostic clinical reasoning. J. Chiropr. Humanit..

[39] Roland H., Brown A., Rousselot A., Freeman N., Wieting J.M., Bergman S., Mondal D. (2022). Osteopathic manipulative treatment decreases hospital stay and healthcare cost in the neonatal intensive care unit. Medicines.

[40] Pizzinato A., Liguoro I., Pusiol A., Cogo P., Palese A., Vidal E. (2022). Detection and assessment of postoperative pain in children with cognitive impairment: A systematic literature review and meta-analysis. Eur. J. Pain.

[41] Nguyen T., Nguyen S., Nguyen T., Doan T., Ngo T. (2025). Pain management and related factors in pediatric patients after congenital heart surgery at Hanoi Heart Hospital. Viet. J. Cardiothorac. Surg..

[42] Gal D.B., Clyde C.O., Colvin E.L., Colyer J., Ferris A.M., Figueroa M.I., Hills B.K., Lagergren S.M., Mangum J., Mann J.L. (2022). Management of postoperative pain after pediatric cardiac surgery: Guidelines. Cardiol. Young.

[43] Küçük Alemdar D., Bulut A., Yilmaz G. (2023). Impact of music therapy and hand massage in the pediatric intensive care unit on pain, fear and stress: Randomized controlled trial. J. Pediatr. Nurs..

[44] Staveski S.L., Boulanger K., Erman L., Lin L., Almgren C., Journel C., Roth S.J., Golianu B. (2018). The impact of massage and reading on children’s pain and anxiety after cardiovascular surgery: A pilot study. Pediatr. Crit. Care Med..

[45] Belsky J.A., Brown A.M. (2024). Investigating the safety and feasibility of osteopathic manipulative medicine in hospitalized children and adolescent young adults with cancer. J. Osteopath. Med..

[46] Diagne P.A., Diop M.S., Ba P.S., Ousmane B.P., Boubacar B.E.H., Mbacké S.E.H., Biram S.E., Mohamed L., Gabriel C.A., Mouhamadou N. (2020). Morbidity and mortality of pediatric cardiac surgery: Retrospective study. Int. J. Cardiovasc. Thorac. Surg..

[47] Kempny A., Dimopoulos K., Uebing A., Diller G.P., Rosendahl U., Belitsis G., Gatzoulis M.A., Wort S.J. (2017). Outcomes of cardiac surgery in congenital heart disease in England, 1997–2015. PLoS ONE.

[48] Soulsby W.D., Lawson E., Okumura M., Pantell M.S. (2023). Socioeconomic factors and hospitalization severity in pediatric lupus. Arthritis Care Res..

[49] Parlar-Chun R., Hafeez Z. (2024). Socioeconomic factors and severity of bronchiolitis hospitalizations. Clin. Pediatr..

[50] Hart S.A., Tanel R.E., Kipps A.K., Hoerst A.K., Graupe M.A., Cassidy S.C., Hlavacek A.M., Clabby M.L., Bush L.B., Zhang W. (2020). Intensive care unit and acute care unit length of stay after congenital heart surgery. Ann. Thorac. Surg..

[51] Cicchitti L., Di Lelio A., Barlafante G., Cozzolino V., Di Valerio S., Fusilli P., Lucisano G., Renzetti C., Verzella M., Rossi M.C. (2020). Osteopathic manipulative treatment in neonatal intensive care units. Med. Sci..

[52] Wernick H., Berkowitz M. (2019). Osteopathic manipulative treatment in the NICU: A systematic review. Acta Sci. Med. Sci..

[53] Lunghi C., Iacopini A., Baroni F., Consorti G., Cerritelli F. (2021). Thematic analysis of attitudes held by a group of Italian osteopaths toward osteopathic evaluation, treatment, and management in the neonatal and pediatric field: A qualitative study. J. Manip. Physiol. Ther..

[54] Sposato N.S., Bjerså K. (2018). Osteopathic manipulative treatment in surgical care: Short review of research publications in osteopathic journals during the period 1990 to 2017. J. Evid. Based Integr. Med..

[55] Posadzki P., Lee M.S., Ernst E. (2013). Osteopathic manipulative treatment for pediatric conditions: A systematic review. Pediatrics.

[56] DeMarsh S., Huntzinger A., Gehred A., Stanek J.R., Kemper K.J., Belsky J.A. (2021). Pediatric osteopathic manipulative medicine: A scoping review. Pediatrics.

[57] Tedeschi R., Giorgi F. (2025). Exploring Manual Interventions for Infantile Colic: A Scoping Review of the Evidence. Children.

